# Higher levels of concern about dieting and moderate-intensity physical activity predict orthorexia nervosa among young adults

**DOI:** 10.1192/j.eurpsy.2022.401

**Published:** 2022-09-01

**Authors:** S. Pardini, J. Szubert, C. Novara, A. Brytek-Matera

**Affiliations:** 1 University of Padova, Department Of General Psychology, Padova, Italy; 2 University of Wroclaw, Institute Of Psychology, Wroclaw, Poland

**Keywords:** orthorexia nervosa, physical activity, Eating Disorders, young adults

## Abstract

**Introduction:**

In some individuals, interest in healthy attitudes and behaviours towards food may show obsessive signs. Preoccupation with ‘healthful’ eating may contribute to orthorexia nervosa (ON) – a strong preoccupation with “healthy eating” manifested by the avoidance of all foods considered by the individual to be “unhealthy”.

**Objectives:**

The objective of the present study was to determine whether disordered eating behaviour, physical activity and self-esteem are predictors of ON in young adults.

**Methods:**

Five hundred fifty-four Polish and Italian university students participated in the present study. Participants were asked to answer the Eating Habits Questionnaire, the Eating Attitudes Test, the International Physical Activity Questionnaire and the Rosenberg Self-Esteem Scale.

**Results:**

Our findings found that higher levels of concern about dieting and moderate-intensity physical activity were related to ON. Particularly, higher levels of concern about dieting, bulimic behaviour and thoughts about food and moderate-intensity physical activity predicted problems associated with healthy eating. Higher levels of concern about dieting, self-esteem as well as self-control of eating and perceived pressure from others to gain weight were associated with knowledge of healthy eating. Whereas, higher levels of concern about dieting with country factor (Poland) predicted feeling positively about healthy eating.

**Conclusions:**

In ON treatment, reduction in symptoms and concerns characteristic of eating disorders and adequate levels of physical activity should be taken into consideration.

**Disclosure:**

No significant relationships.

